# ZnO-based micromotors fueled by CO_2_: the first example of self-reorientation-induced biomimetic chemotaxis

**DOI:** 10.1093/nsr/nwab066

**Published:** 2021-04-20

**Authors:** Fangzhi Mou, Qi Xie, Jianfeng Liu, Shengping Che, Lamya Bahmane, Ming You, Jianguo Guan

**Affiliations:** State Key Laboratory of Advanced Technology for Materials Synthesis and Processing, International of Materials Science and Engineering, Wuhan University of Technology, Wuhan 430070, China; State Key Laboratory of Advanced Technology for Materials Synthesis and Processing, International of Materials Science and Engineering, Wuhan University of Technology, Wuhan 430070, China; State Key Laboratory of Advanced Technology for Materials Synthesis and Processing, International of Materials Science and Engineering, Wuhan University of Technology, Wuhan 430070, China; State Key Laboratory of Advanced Technology for Materials Synthesis and Processing, International of Materials Science and Engineering, Wuhan University of Technology, Wuhan 430070, China; State Key Laboratory of Advanced Technology for Materials Synthesis and Processing, International of Materials Science and Engineering, Wuhan University of Technology, Wuhan 430070, China; State Key Laboratory of Advanced Technology for Materials Synthesis and Processing, International of Materials Science and Engineering, Wuhan University of Technology, Wuhan 430070, China; State Key Laboratory of Advanced Technology for Materials Synthesis and Processing, International of Materials Science and Engineering, Wuhan University of Technology, Wuhan 430070, China

**Keywords:** micro/nanomotors, zinc oxide, carbon dioxide, self-propulsion, chemotaxis

## Abstract

Synthetic chemotactic micro/nanomotors are envisioned to actively ‘seek out’ targets by following specific chemicals, but they are mainly powered by bioincompatible fuels and only show pseudochemotaxis (or advanced chemokinesis) due to their weak self-reorientation capabilities. Here we demonstrate that synthetic ZnO-based Janus micromotors can be powered by the alternative biocompatible fuel of CO_2_, and further provide the first example of self-reorientation-induced biomimetic chemotaxis using them. The ZnO-based micromotors are highly sensitive to dissolved CO_2_ in water, which enables the corrosion of ZnO to continuously occur by providing H^+^ through hydration. Thus, they can autonomously move even in water exposed to air based on self-diffusiophoresis. Furthermore, they can sense the local CO_2_ gradient and perform positive chemotaxis by self-reorientations under the phoretic torque. Our discovery opens a gate to developing intelligent micro/nanomotors powered by, and sensitive to, biocompatible atmospheric or endogenous gaseous chemicals for biomedical and environmental applications.

## Teaser

Synthetic ZnO-based micromotors can autonomously move in water by acquiring CO_2_ from air, and perform positive chemotaxis following the local CO_2_ gradient by self-reorientations under the electro-osmotic viscous torque.

## INTRODUCTION

Biological chemotaxis refers to the ability of those living organisms that are of an advanced level of evolution to move toward or away from specific chemicals [1,2]. To achieve chemotaxis, the living organisms are equipped with delicately coordinated sensing, signal processing and propelling systems, endowing them with the capability to adjust their motion direction on demand by evaluating the direction of chemical gradients at different times (e.g. the temporal comparison adopted by *Escherichia coli*) or perceiving signal intensity differences across their body (e.g. the spatial comparison adopted by neutrophils) [3,4]. Chemotaxis not only regulates the development of multicellular systems such as tissue development and cancer metastasis, but also enables many unicellular organisms to find nourishments, avoid toxins and coordinate collective motions [5–7].

Inspired by biological chemotaxis, researchers strive to create synthetic chemotactic micro/nanomotors (MNMs). They are envisioned to achieve self-navigation and self-targeting in complex or dynamic environments by actively following specific chemical cues, and thus may bring revolutionary changes to targeted drug delivery, microsurgeries, microfactories, etc [8]. Nonetheless, synthetic MNMs are generally too small to incorporate sophisticated sensing-processing-propelling systems and have a low sensitivity to chemical gradients. Thus, they only show a pseudochemotaxis (or macroscopic chemotaxis and advanced chemokinesis) based on a purely statistical effect rather than chemotaxis of individual MNMs because of the stochastic nature in motion for each individual [8,9]. With the pseudochemotaxis, they show negligible reorientation capabilities, and can only accumulate near (or away from) the chemoattractant (or chemorepellent) source, or shift toward (or away from) its stream in microfluidic channels due to their spatial-dependent mobility (orthokinesis) in the local chemical gradient field [8,10–13]. This statistically average movement uphill (or downhill) to a chemical gradient based on orthokinesis undermines the self-targeting performance of MNMs, and is far from the biological chemotaxis found in nature. On the other hand, chemically powered MNMs have been predicted, in theory, to achieve chemotaxis through their self-reorientation based on the spatial comparison of chemical signal intensity across their bodies, like neutrophils [14,15], but it has not been experimentally confirmed.

In this work, we demonstrate ZnO-based Janus micromotors (MMs) powered by an alternative fuel of CO_2_, and further provide the first example of self-reorientation-induced biomimetic chemotaxis using them. The ZnO-based MMs can autonomously move based on electrolyte self-diffusiophoresis, even in water exposed to air, as the ZnO component in the MMs is highly sensitive to CO_2_ dissolved in water, which continuously produces H^+^ through hydration to react with ZnO. In this way, the MMs can sense local CO_2_ signals, and exhibit a biomimetic chemotactic behavior toward the CO_2_ source due to their dynamic reorientations under a phoretic torque. The proposed alternative fuel of CO_2_, which is a constituent gas in the air and a major product of cell respiration [16,17], has remarkable advantages, including sustained remote accessibility, low cost and excellent biocompatibility. With the excellent biomedical activities of ZnO [18], the intelligent chemotactic behaviors promise that the ZnO-based MMs will ‘seek out’ specific cells or pathogen microorganisms by tracking metabolic CO_2_ signals emitted from them, and execute targeted biomedical and environmental operations.

## RESULTS AND DISCUSSION

**Figure 1.**
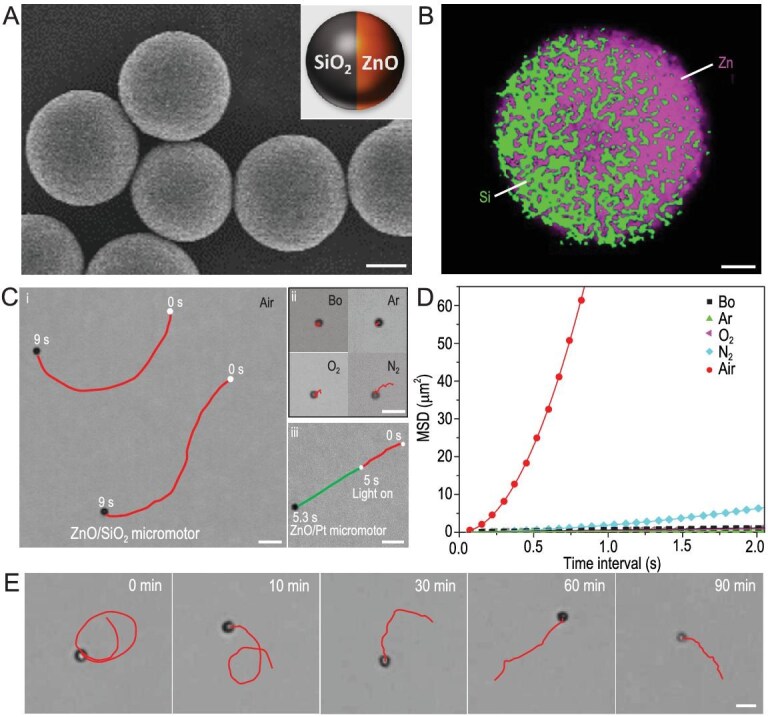
Characterization and self-propulsions of the ZnO-based MMs. (A) SEM image of the ZnO/SiO_2_ MMs. Scale bar, 1 μm. The inset shows the schematic illustration of their Janus structure. (B) EDX mapping of Zn and Si elements in a ZnO/SiO_2_ MM. Scale bar, 500 nm. (C) Self-propulsions of ZnO-based MMs in different water media. (i and ii) Trajectories of ZnO/SiO_2_ MMs in 9 s in the (i) air-exposed water (Air) and (ii) CO_2_-eliminated water, including the freshly boiled water (Bo), Ar-gassed water (Ar), O_2_-gassed water (O_2_) and N_2_-gassed water (N_2_). Scale bars, 10 μm. (iii) Trajectories of a ZnO/Pt MM in the air-exposed water without (red curve) and with (green curve) UV irradiation. Scale bars, 5 μm. (D) MSD of the MMs in different water media. Solid color lines are fitting curves using a quadratic function. (E) Self-propulsion of a typical ZnO/SiO_2_ MM within a lifetime of 90 min. Red curves depict its trajectories in 9 s at different time intervals. Scale bar, 5 μm.

To experimentally achieve the biomimetic chemotaxis of artificial MNMs, we at first prepared ZnO-based MMs by sputtering a thin layer of SiO_2_ on the exposed surface of hexagonal wurtzite ZnO microspheres (Supplementary Fig. 1A and B), as illustrated in Supplementary Fig. 2. The scanning electron microscope (SEM) observation indicates that the prepared ZnO/SiO_2_ MMs have a relatively uniform spherical shape and an average diameter (*d*) of 2.5 μm (Fig. 1A). Energy dispersive X-ray (EDX) mapping of Zn and Si elements confirms that the ZnO/SiO_2_ MMs have a typical Janus structure (the inset in Fig. 1A, and Fig. 1B). The thickness of the sputtered SiO_2_ layer is ∼150 nm, as verified by the high-contrast SEM observation (Supplementary Fig. 3A) and line-scanning EDX analysis of Zn and Si elements (Supplementary Fig. 3B) on a typical ZnO/SiO_2_ MM. Zeta potential test shows that the ZnO microspheres before and after coating SiO_2_ have a similar negative surface charge of 24.1 and −23.7 mV, respectively.

When the ZnO/SiO_2_ MMs were put into water previously exposed to air (air-exposed water, see ‘Materials’ section in the online Supplementary Data), they exhibited an autonomous motion with an average speed (*v*) of 7.8 μm/s (Fig. 1C(i) and Supplementary Video 1). As ZnO is poorly reactive or soluble in pure water [19], the self-propulsion of the MMs was attributed to atmospheric CO_2_ because it has a much higher solubility (34 mM at 25^o^C and 1 atm) in water compared to other major constituent gases (such as N_2_, O_2_ and Ar) [20]. To confirm the key role of CO_2_ in the self-propulsion of the ZnO/SiO_2_ MMs, we investigated their motions in CO_2_-eliminated water (see ‘Materials’ section in the online Supplementary Data), including freshly boiled water (pH = 7.54), Ar-gassed water (pH = 7.46), O_2_-gassed water (pH = 7.36) and N_2_-gassed water (pH = 6.67), respectively (Supplementary Video 1). As shown in Fig. 1C(ii) and D, they show an extremely slow motion in the N_2_-gassed water and only Brownian motions in other CO_2_-eliminated water, with a translational *v* and diffusivity (*D*) lower than 1.3 μm/s and 0.14 μm^2^/s, respectively. The translational *v* and *D* were extracted by fitting their mean square displacements (MSDs, Fig. 1D) using a quadratic function according to the previous report [21].

The SiO_2_ cap on the ZnO/SiO_2_ MMs only acts as a passive layer to break the symmetry of the surface reaction of ZnO. This can be verified by the fact that the ZnO microspheres only exhibit Brownian motions in the air-exposed water due to isotropic surface reactions (Supplementary Fig. 4), and the MM can still be self-propelled in the same condition if the SiO_2_ cap is replaced by a Pt cap (Fig. 1C(iii)). The ZnO/SiO_2_ MMs showed similar motions in low-energy red light (20.2 mW/cm^2^) and high-energy UV light (300 mW/cm^2^) (Supplementary Fig. 5), while the ZnO/Pt MMs were accelerated under the irradiation of UV light because of their high photocatalytic activity (Supplementary Video 2 and Fig. 1C(iii)). This suggests that light illumination has a negligible contribution to the self-propulsion of ZnO/SiO_2_ MMs. Air-exposed water has a CO_2_ concentration (*C*_CO_2__) of 8.54 μM (pH = 5.72), which is three orders lower than the critical concentration of the H_2_O_2_ fuel (∼60 mM) required to power traditional catalytic Pt-based MNMs [22]. This suggests that the MMs are highly sensitive to dissolved CO_2_ in water and can be powered at an ultralow level of the chemical fuel. Moreover, the ZnO/SiO_2_ MMs exhibit a long lifetime of ∼90 min (Fig. 1E), which is higher than that of the active metal-based MMs (∼5 min), catalytic MnO_2_ MMs (≤40 min) and some enzyme-based MMs (≤40 min) [23–25].

CO_2_ exists as a stable and inert gas in air, but can remotely diffuse into various aqueous media to participate in a number of reactions after hydration, such as acidic corrosion [17,26–29]. In detail, when water is exposed to air, CO_2_ molecules, which account for ∼0.04% of the air in volume [30], can enter into the water through the interface with the atmosphere, and are partially hydrated into H_2_CO_3_, which then dissociates into HCO$_3^{-}$ and H^+^, as described by equation (1) [31]:

(1) \begin{equation*}{\mathrm{C}}{{\mathrm{O}}_2} + {{\mathrm{H}}_2}{\mathrm{O}} \mathbin{\lower.3ex\hbox{$\buildrel\textstyle\leftharpoonup\over {\smash{\rightharpoondown}}$}} {{\mathrm{H}}_2}{\mathrm{C}}{{\mathrm{O}}_3} \mathbin{\lower.3ex\hbox{$\buildrel\textstyle\leftharpoonup\over {\smash{\rightharpoondown}}$}} {{\mathrm{H}}^ + } + {\mathrm{HCO}}_3^{ -}.
\end{equation*}

Afterwards, the ZnO-based MMs react with the released H^+^ according to equation (2) [19]:

(2) \begin{equation*}2{{\mathrm{H}}^ + } + {\mathrm{ZnO}} \to {\mathrm{Z}}{{\mathrm{n}}^{2 + }} + {{\mathrm{H}}_2}{\mathrm{O}}.\end{equation*}

The acidity constant (*K_a_*) of the dissolved CO_2_ molecules is 4.3 × 10^–7^ M at room temperature, indicating that only a small amount of them are converted into H^+^ and HCO$_3^{-}$. In this way, the dissolved CO_2_ stayed as molecules can continuously supply H^+^ ions through the local equilibrium reaction (equation (1)) when H^+^ is consumed by ZnO, and thus CO_2_ is considered as the chemical fuel powering the ZnO-based MMs according to an overall reaction expressed as equation (3):

(3) \begin{equation*}{\mathrm{ZnO}} + 2{\mathrm{C}}{{\mathrm{O}}_2} + {{\mathrm{H}}_2}{\mathrm{O}} \to {\mathrm{Z}}{{\mathrm{n}}^{2 + }} + 2{\mathrm{HCO}}_3 ^{-}.
\end{equation*}

**Figure 2.**
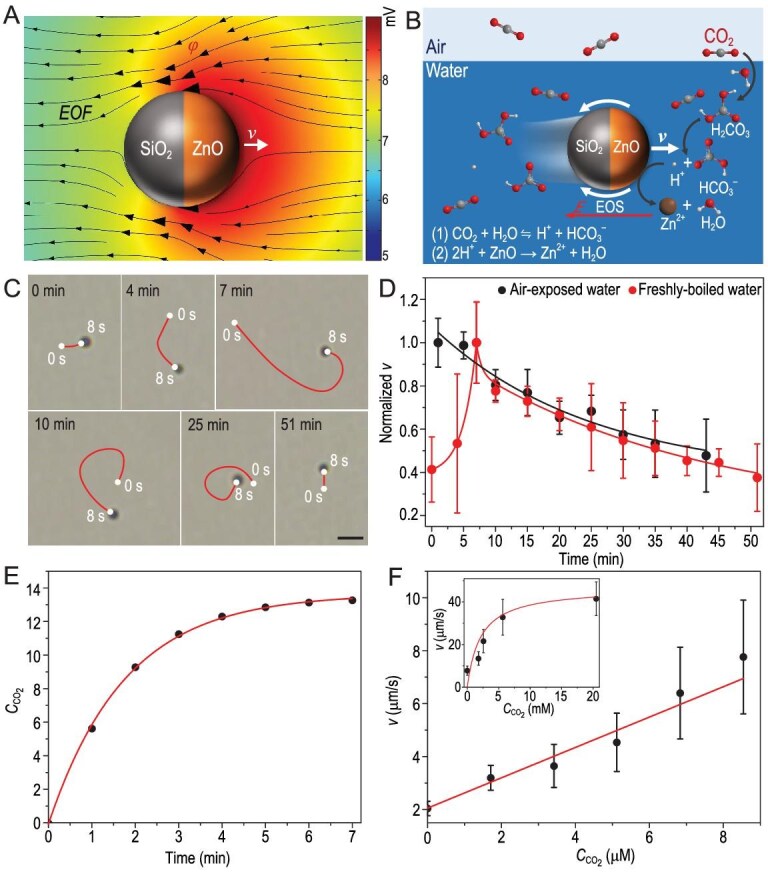
The self-propulsion mechanism of the ZnO/SiO_2_ MMs. (A) Numerical simulations of the electric potential (*ϕ*, the color background) and the electro-osmotic flow (EOF, black streamlines with arrows) around a ZnO/SiO_2_ MM. The EOF is initiated by the surface electro-osmotic slip (EOS) in its electric double layer. (B) Schematic illustration of the propulsion mechanism of the ZnO/SiO_2_ MMs. (C) Trajectories of the MMs in freshly boiled water at different time intervals when exposed to air. Scale bar, 5 μm. (D) Time-dependent normalized *v* of the MMs in a film of freshly boiled water when exposed to air (red dots) and the air-exposed water (black dots). Solid red and black curves are fitting results using exponential functions. (E) The simulated *C*_CO_2__ as a function of time in a CO_2_-eliminated water film (160 μm in thickness) when exposed to air. (F) The *v* of the MMs at different *C*_CO_2__ in water. The red line is the linear fitting line of the experimental data in the low *C*_CO_2__ regime (0–8.54 μM), and the red curve in the inset is the fitting result according to the Michaelis-Menten kinetics in the high *C*_CO_2__ regime (8.54 μM to 20.5 mM).

Due to the different *D* of the released ionic products of HCO$_3^{-}$ (*D* = 1.185 × 10^–9^ m^2^/s) and Zn^2+^ (*D* = 0.703 × 10^–9^ m^2^/s) from the ZnO/SiO_2_ MMs according to equation (3), HCO$_3^{-}$ would diffuse faster than Zn^2+^ [32,33], leading to an uneven distribution of these ions in the area near the exposed ZnO surface (Supplementary Fig. 6A and B). To maintain electroneutrality, a local diffusioelectric field (*E*) arises to slow down the faster-diffusing ion, and speed up the slower ion [34], as verified by the simulated electric potential *ϕ* depicted in the color background of Fig. 2A and the local *E* shown in Supplementary Fig. 6C (black triangles). This local *E* then induces an electro-osmotic slip (EOS) [35] in the electric double layer of the negatively charged MM (*ζ* = −23.7 mV). The simulated flow field (EOF) initiated by the surface EOS around a ZnO/SiO_2_ MM is illustrated as black streamlines with arrows in Fig. 2A, revealing that the MMs are self-propelled based on electrolyte self-diffusiophoresis with the ZnO end forward. This propulsion direction was further experimentally verified by the close observation of a partially etched ZnO/SiO_2_ MM and the barely etched SiO_2_/ZnO MMs, both of which have a distinguishable ZnO end at a high magnification under an optical microscope (Supplementary Figs 7 and 8, Supplementary Video 3). The detailed propulsion mechanism of the ZnO/SiO_2_ MM fueled by the dissolved atmospheric CO_2_ in water is illustrated in Fig. 2B.

From the propulsion mechanism (Fig. 2B), it is expected that the ZnO-based MMs can still acquire fuel from the air even if no CO_2_ was pre-dissolved in water. To verify this assumption, we recorded the motion behaviors of the ZnO/SiO_2_ MMs in a thin film (∼160 μm in thickness) of the freshly boiled water over time when exposed to air (Fig. 2C and Supplementary Video 4). Differently to the MMs in the air-exposed water film (*C*_CO_2__ = 8.54 μM), which show a decreasing *v* over time (the black curve with dots in Fig. 2D), those in the CO_2_-eliminated water display an increasing *v* in the first 7 min (the red curve with dots in Fig. 2D). This behavior can be verified by the different lengths of trajectories of a typical MM in 8 s (Fig. 2C) and the statistic normalized *v* of the MMs (Fig. 2D) at different intervals within 7 min. This increase in *v* reveals that atmospheric CO_2_ molecules can diffuse into the water as fuel to power the MMs in a sustainable and rapid manner, as verified by the numerical results of the increasing *C*_CO_2__ (Fig. 2E and Supplementary Fig. 9A) and H^+^ concentration (${C_{{{\mathrm{H}}^ + }}}$, Supplementary Fig. 9B) over time when the water film is exposed to air. In the following 7–51 min, the *v* of the MMs decreases gradually due to the decreasing reaction rate of ZnO and the increasing ion concentration in the water film. With the increase of *C*_CO_2__ from 0 to 8.54 μM, the *v* of the ZnO/SiO_2_ MMs increases from 2.0 to 7.8 μm/s (Fig. 2F and Supplementary Video 5). By bubbling water with pure CO_2_ gas, a higher *C*_CO_2__ compared to that in the air-exposed water can be obtained, and the *v* of MMs can reach 41.5 μm/s when *C*_CO_2__ increases to 20.5 mM (the inset in Fig. 2F and Supplementary Video 5). The fitting results show that the *v* of the MMs increases linearly in the low *C*_CO_2__ regime from 0 to 8.54 μM (red line in Fig. 2F), and gradually reaches to a plateau in the high *C*_CO_2__ regime (8.54 μM–20.5 mM) following Michaelis-Menten kinetics (red curve in the inset of Fig. 2F). These results reveal that the ZnO/SiO_2_ MMs are of a high sensitivity to *C*_CO2_, especially in the low *C*_CO_2__ regime. The sensitivity can be quantified by the rate of the speed change of the ZnO/SiO_2_ MMs to *C*_CO_2__, which has a value of 0.57 μm/(μM·s) (the slope of the red line in Fig. 2F) in the low *C*_CO_2__ regime, about five orders of magnitude higher than that of common H_2_O_2_-fueled MMs (2.72 × 10^–6^ μm/(μM·s)) [36]. The appearance of the plateau of *v* at a high *C*_CO_2__ of 20.5 mM indicates that the maximum speed is limited by the maximum reaction rate depending on total surface reactive sites.

**Figure 3.**
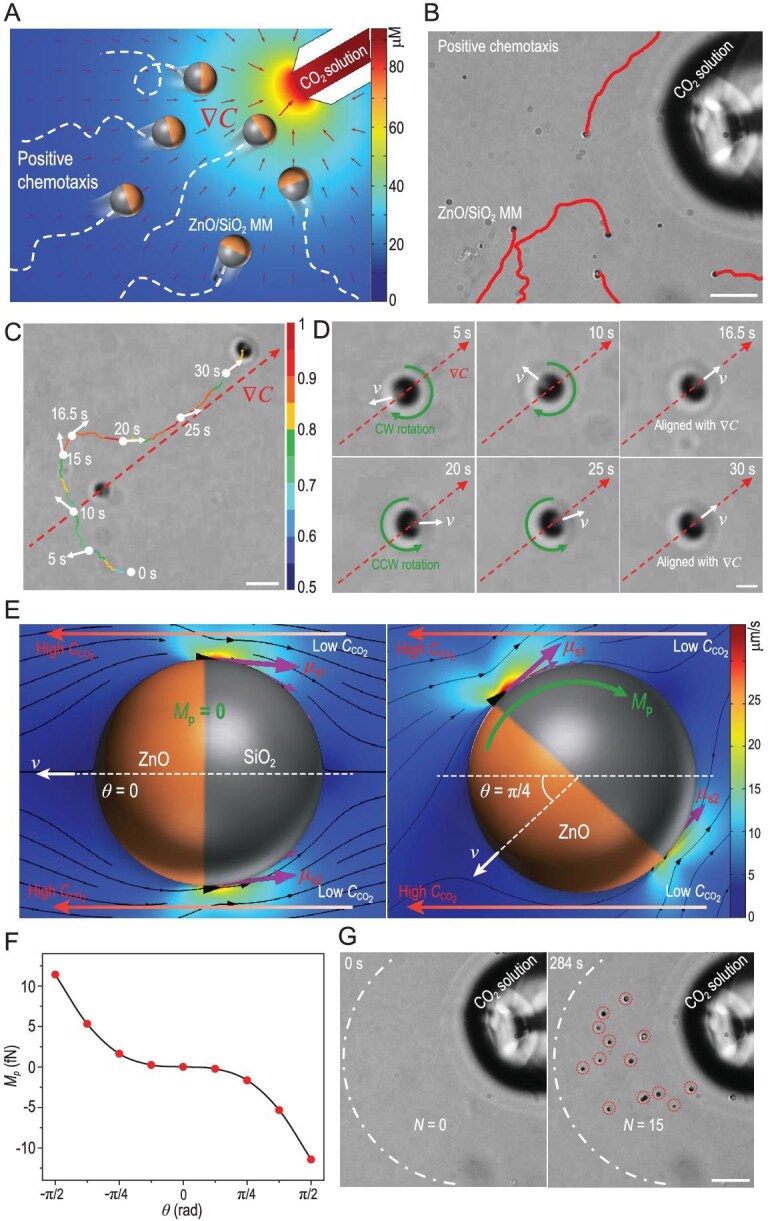
Chemotaxis of the ZnO/SiO_2_ MMs. (A) Schematic illustration of the positive chemotaxis of the ZnO/SiO_2_ MMs. The color background depicts numerical simulations of *C*_CO_2__ gradient ($\nabla C$) near a micropipette (inner *d*, 20 μm) filled with a CO_2_ solution (*C*_CO_2__ = 93.7 μM). (B) Trajectories of the MMs moving toward the CO_2_ source. Scale bar, 20 μm. (C) The color trajectory of a slightly etched ZnO/SiO_2_ MM approaching the CO_2_ source, in which the colors represent the normalized magnitude of *v* of the MM. Scale bar, 5 μm. (D) The reorientations of the ZnO/SiO_2_ MM shown in (C) at different times by clockwise (CW) or counterclockwise (CCW) rotations. Scale bar, 2 μm. (E) Numerical simulations of the EOSs (purple arrows) on a ZnO/SiO_2_ MM and the resulted flow fields (black streamlines with black arrows) when it aligns with $\nabla C$ (*θ* = 0, the left panel) and deviates with it in a *θ* of π/4 (the right panel), respectively. The color background depicts the magnitude of the flow velocity. The green arrow represents the phoretic torque (*M*_p_) induced by the unbalanced EOSs. (F) The calculated *M*_p_ at different *θ*. (G) Time-lapse images displaying the change of *N* near the CO_2_ source. Scale bar, 20 μm.

Benefiting from the high sensitivity to *C*_CO_2__, the ZnO/SiO_2_ MMs can sense a local CO_2_ gradient and exhibit positive chemotaxis toward a local CO_2_ source in water (Fig. 3A). When a micropipette filled with a CO_2_ solution (*C*_CO2_ = 93.7 μM) was introduced to the water medium (*C*_CO_2__ = 0.2 μM), a gradient field of *C*_CO_2__ ($\nabla C$) is formed (the color background in Fig. 3A). In this gradient field, the ZnO/SiO_2_ MMs swarm toward the micropipette, in analogy to the positive chemotaxis of living organisms in nature (Fig. 3B and Supplementary Video 6). The average chemotactic velocity, defined as the net displacement of the ZnO/SiO_2_ MMs divided by time when they are approaching the region close to (∼20 μm) the CO_2_ source, is 0.57 μm/s. In contrast, passive SiO_2_ microspheres (*d* = 2 μm and *ζ* = −18.5 mV) show no chemotactic motion in the same gradient field (Supplementary Fig. 10 and Supplementary Video 7), indicating that the MMs were actively swimming toward the CO_2_ source, and the possible passive diffusiophoretic attraction generated by the gradient field is negligible.

To quantify the degree of chemotaxis, we have analyzed the chemotaxis index (*CI*) of ZnO/SiO_2_ MMs, which was defined as the ratio of the displacement in the direction of the chemical gradient to the total migration distance (i.e. as the cosine of the angle *θ* of the MM’s motion direction with the chemical gradient) [37]. The average *CI* is 0.21, suggesting that they, in general, move uphill to the chemical gradient. Due to the persistent ZnO-forward configurations during the propulsion (Supplementary Figs 6 and 7), the self-reorientation dynamics of the ZnO/SiO_2_ MMs can be characterized by the changes of *CI* and the angle *θ* over time. When *CI >* 0 (i.e. $0 \le | \theta | < {\mathrm{\pi }}/2$), the ZnO/SiO_2_ MMs prefer to move up the gradient, while they tend to move down the gradient if *CI <* 0 (i.e. ${\mathrm{\pi }}/2 < | \theta | \le {\mathrm{\pi }}$). The *CI* and |*θ*| of four typical ZnO/SiO_2_ MMs versus time (Supplementary Fig. 11) suggest that they move up the gradient for a longer time than moving down. The longer upgradient motion time implies that the ZnO/SiO_2_ MMs prefer to move with an orientation along the upgradient direction even though their motion direction was seriously randomized by the Brownian rotational diffusions.

To further demonstrate the biomimetic chemotactic behavior, the instantaneous *v*, orientation and rotation of a slightly etched ZnO/SiO_2_ MM, in which the slightly etched ZnO end was distinguishable under an optical microscope, were recorded and analyzed when it was approaching the CO_2_ source (Fig. 3C and D). The ZnO/SiO_2_ MM shows an increasing *v* when approaching the CO_2_ source because of the increasing *C*_CO_2__ (i.e. orthokinesis of the motor [38]), as verified by the normalized instantaneous *v* in 0–15 s and 15–30 s (Fig. 3C), respectively. In addition, when the ZnO/SiO_2_ MM deviates with $\nabla C$ because of the strong perturbations of Brownian randomizations, it dynamically reorientates itself by clockwise or counterclockwise rotations (Fig. 3D). When the MM moves with an orientation down $\nabla C$, it rotates clockwise gradually and aligns with $\nabla C$ (0–16.5s in Fig. 3D). After a sharp turn (16.5–20 s in Fig. 3D), the MM deviates with $\nabla C$ again, and then gradually aligns with $\nabla C$ by a counterclockwise rotation (20–30 s in Fig. 3D). This self-reorientation behavior bears resemblance to that of chemotactic microorganisms which show a ‘memory’ of the direction of the chemical gradient and move up the gradient of chemoattractant in a ‘deviating-rectifying’ manner. This is the first experimental evidence showing that synthetic MNMs can achieve chemotaxis by dynamic self-reorientations in a gradient field [8]. After reaching the area close to the tip of the micropipette (CO_2_ source), the MMs were wandering in this area with no preferred directions, as depicted by the curved trajectory of the slightly etched MM in 34–163 s in Supplementary Fig. 12, so that they could accumulate near the CO_2_ source (Supplementary Video 6).

To decipher the chemotactic behavior, we have simulated the flow field around the ZnO-based MM when a local CO_2_ source is introduced, as shown in Fig. 3E. The results show that, if the axis of the MM is aligned with the CO_2_ gradient, EOSs (e.g. *u*_s1_ and *u*_s2_ in the left panel in Fig. 3E, *u*_s1_ = *u*_s2_) across the axis of the MM are symmetrical because of symmetric surface reactions, and the MM migrates up the gradient at this condition. However, due to the strong rotational Brownian diffusion, the symmetry axis of the MM is constantly deviating from $\nabla C$. When misaligned with $\nabla C$ with an angle of *θ*, the ZnO surface close to the CO_2_ source has a higher reaction rate (due to the higher *C*_CO_2__ (or ${C_{{{\mathrm{H}}^ + }}}$)) than the surface further away from the source, resulting in the asymmetric distribution of chemical products and thus unbalanced EOSs across the motor axis (e.g. *u*_s1_ and *u*_s2_ in the right panel of Fig. 3E, *u*_s1_ > *u*_s2_). The unbalanced EOSs then produce a phoretic torque (*M*_p_) to induce rotation of the MM until its axis is aligned with $\nabla C$ (the right panel of Fig. 3E). With the increasing degree of deviation, the self-EOSs across the motor axis become more unbalanced, and thus *M*_p_ increases accordingly for reorientation, as verified by the increasing *M*_p_ with a varying *θ* from 0 to -π/2 and π/2 (Fig. 3F). Hence, the chemotactic behavior of the MM is attributed to dynamic self-reorientations under the phoretic torque. Due to their self-reorientations, they show a higher efficiency in chemotaxis compared to the developed (pseudo)chemotactic MNMs, which usually take hours to aggregate near the chemical source [10,39,40]. As shown in Fig. 3G, the MMs swarm to the chemical source in several minutes, as demonstrated by the increasing number (*N*) of MMs from 0 to 15 within 284 s in a sector region close to (80 μm) the CO_2_ source.

CO_2_ not only exists in the air and various aqueous media (such as natural waters and biological media), but also can be produced and secreted by cells or pathogenic microorganisms during their oxidative metabolism [41–44]. For instance, the CO_2_ tension (*P*_CO_2__) of the intracellular and extracellular fluids is ∼50 and 46 mmHg, indicating that there is a high *C*_CO_2__ (∼20.8 and 19.3 mM together with the chemically bound CO_2_) and a steep *C*_CO_2__ gradient near tissue cells (e.g. ∼3.3 × 10^4^ mol/m^4^ at a distance of 20 μm to the cell surface), respectively [45]. On the other hand, ZnO micro/nanomaterials are of low toxicity and good biodegradability and have shown great potential in biomedical applications due to their excellent anti-cancer, anti-diabetic, anti-bacterial and anti-inflammatory activities [18]. Thus, the chemotactic ZnO-based MMs with proper surface modifications (e.g. polyelectrolyte ion-tolerance coating [46]) are expected to act as biomimetic microrobots, ‘seeking out’ specific cells or pathogenic microorganisms by actively tracking their extracellular CO_2_ signals, thereby executing targeted biomedical and environmental operations.

## CONCLUSION

In conclusion, we have demonstrated that ZnO-based MMs can be powered by CO_2_ fuels and perform intelligent positive chemotaxis by self-reorientations. We analyzed the propulsion mechanism of ZnO-based MMs and confirmed that it was the result of electrolyte self-diffusiophoresis based on the corrosion of the ZnO end by H^+^ continuously supplied from the dissolved CO_2_. Benefiting from their high sensitivity to local CO_2_ signals, the ZnO-based MMs can perform a biomimetic chemotactic behavior toward the CO_2_ source due to their dynamic self-reorientations in the chemical gradient field. The self-reorientation of the MM is attributed to the phoretic torque produced by the unbalanced EOSs across the MM axis when it is misaligned with the chemical gradient. Due to the readily accessible biocompatible CO_2_ fuel and intelligent chemotaxis, ZnO-based MMs have the potential to autonomously move in a wide range of aqueous media and execute targeted biomedical and environmental operations by actively tracking respiratory CO_2_ signals emitted from cells or pathogenic microorganisms.

## METHODS

### Preparation of ZnO-based MMs

The ZnO microspheres were synthesized according to the previous report [47]. To prepare ZnO/SiO_2_ MMs, the ZnO microspheres on the glass slide were partially coated with a SiO_2_ nanolayer by magnetron sputtering for 40 min (JCP500, Beijing Technol Science Co., Ltd, China). The substrate was heated to 100^o^C and rotated at a speed of 30 r/min during the sputtering process. In addition, ZnO/Pt MMs and SiO_2_/ZnO MMs were also prepared by sputtering Pt on the ZnO microspheres on the glass slide for 30 s and a ZnO layer on monodispersed SiO_2_ microspheres (2 μm) for 60 min, respectively.

### Self-propulsion and chemotaxis

To observe the self-propulsion of ZnO/SiO_2_ MMs, a 20 μL aqueous suspension of the MMs (0.03 mg/mL) was dropped into a Petri dish (*d* = 35 mm) with 2 ml water. The self-propulsion of the MMs was observed and recorded through an inverted optical microscope (Leica DMI 3000B). All videos were analyzed using ImageJ and Video Spot Tracker V08.01 software. More than 10 MMs were analyzed to obtain the statistical result. To investigate the MM’s chemotaxis, a micropipette (inner *d*, 20 μm) filled with a CO_2_ solution (*C*_CO_2__ = 93.7 μM) was fixed on the holder of a high-precision micromanipulator (Leica Microsystems, Germany) and then put in a water droplet (50 μL, pH = 6.52) with the ZnO/SiO_2_ MMs (0.003 mg/mL).

### Numerical simulations

The simulations were performed by using the diffusions, electrostatics and creeping flow modules of COMSOL Multiphysics software [21]. Governing equations for numerical simulations are given in the online Supplementary Data. The simulation model was built up by immersing a ZnO/SiO_2_ MM in the middle of a cylindric cell (radius *r* = 50 μm, height *h* = 100 μm) filled with the air-exposed water (*C*_CO_2__ = 8.54 μM). The fluxes (*J*) of Zn^2+^, H^+^ and HCO$_3^{-}$ from the ZnO surface were set as 0.05, −0.1 and 0.1 mmol m^–2^ s^–1^, respectively. The *D* of Zn^2+^, CO_2_, H^+^ and ${{\rm HCO}_3^-}$ were set as 0.70 × 10^–9^, 1.77 × 10^–9^, 9.31 × 10^–9^ and 1.19 × 10^–9^ m^2^/s, respectively. The *ζ* of the MM was set as −23.7 mV. To simulate the reorientation of the MM during chemotaxis, a CO_2_ source (*C*_CO_2__ = 93.7 μM) was added in the above model and placed at the left boundary with an *r* of 10 μm and a distance of 50 μm to the center, and *J* of Zn^2+^ and ${{\rm HCO}_3^-}$ at each point of the exposed ZnO surface was set to be proportional to the local ${C_{{{\mathrm{H}}^ + }}}$. To simulate the diffusion kinetics of atmospheric CO_2_ (0.04 vol.% in the air) into water through the interface with the atmosphere, a simulation model of a water film (160 μm in thickness) was built and the classical two-film theory was used [48], in which the mass transfer coefficient (*k*) of CO_2_ in the water was set to be 2 μm/s [49], and the equilibrium concentration (C*) of CO_2_ at the interface was calculated to be 1.2 × 10^–5^ M according to Henry's Law equation.

## Supplementary Material

nwab066_Supplemental_Files
