# Supplementary material for: ZnO-based micromotors fueled by CO2: the first example of self-reorientation-induced biomimetic chemotaxis
Source: Natl Sci Rev. 2021 Apr 20;8(11):nwab066. doi: 10.1093/nsr/nwab066 (PMC8645024; doi:10.1093/nsr/nwab066)
Supplement: nwab066_Supplemental_Files [file nwab066_supplemental_files.zip › Supplementary data.docx]

**Supplementary Data**

**ZnO-Based Micromotors Fueled by CO_2_: The First Example of Self-Reorientation-Induced Biomimetic Chemotaxis**

*Fangzhi Mou*†*, Qi Xie*†*, Jianfeng Liu, Shengping Che, Lamya Bahmane, Ming You, Jianguo Guan**

State Key Laboratory of Advanced Technology for Materials Synthesis and Processing, International School of Materials Science and Engineering, Wuhan University of Technology

*Corresponding Author. Email: [guanjg@whut.edu.cn](mailto:guanjg@whut.edu.cn)

Phone: +86-027-87218832

Fax: +86-027-87879468

†Equally contributed to this work.

**Abstract:** Synthetic chemotactic micro/nanomotors are envisioned to actively ‘seek out’ targets by following specific chemicals, but they are mainly powered by bioincompatible fuels and only show pseudochemotaxis (or advanced chemokinesis) due to their weak self-reorientation capabilities. Here we demonstrate that synthetic ZnO-based Janus micromotors can be powered by an alternative biocompatible fuel of CO_2_, and further provide the first example of self-reorientation-induced biomimetic chemotaxis using them. The ZnO-based micromotors are highly sensitive to the dissolved CO_2_ in water, which enables the corrosion of ZnO to continuously occur by providing H^+^ through hydration. Thus, they can autonomously move even in the water exposed to air based on self-diffusiophoresis. Furthermore, they can sense local CO_2_ gradient and perform positive chemotaxis by self-reorientations under the electroosmotic viscous torque. Our discovery opens a gate to develop intelligent micro/nanomotors powered by and sensitive to biocompatible atmospheric or endogenous gaseous chemicals for biomedical and environmental applications.

**Keywords:** micro/nanomotors, zinc oxide, carbon dioxide, self-propulsion, chemotaxis

**SUPPLEMENTARY METHODS**

**Materials**

Zinc nitrate hexahydrate (Zn (NO_3_)_2_·6 H_2_O) and triethanolamine (C_6_H_15_NO_3_) used in the study were purchased from Sinopharm Chemical Reagent and Shanghai Chemical Reagent, respectively. These reagents were used as received without further purification. Deionized (DI) water of 18.2 MΩ cm resistivity from Milli Q Millipore (Merck, USA) was stored in a wash bottle for 24 h and then used as the air-exposed water, and it had a pH of 5.72. The CO_2_-eliminated water was obtained by heating and boiling the DI water for 20 minutes and then cooling to room temperature, or continuously bubbling it with high-purity Ar, O_2_ or N_2_ gas for 40 min at a gas flow rate of 20 ml/min, respectively. The CO_2_ solutions with different CO_2_ concentrations (*C*_CO2_) were obtained by diluting the air-exposed water with the CO_2_-eliminated water (the boiled water) or bubbling the water with pure CO_2_ gas for a different time. To avoid possible impurities which may be introduced to water when bubbling, a Midisart® small-scale venting filter (Sartorius Stedim, Germany) was used in the venting pipe. *C*_CO2_ in various waters was calculated from the measured pH value of the media according to Equation 1,

$$C_{CO2}=\frac{{10}^{-2\mathrm{pH}}}{K_{a}}$$

Here, *K*_a_ is the acidity constant of the dissolved CO_2_ molecules in the water at room temperature (4.3×10^-7^ M). To obtain the low-energy red light, the Fluor-Protection Shield for DMI-Series (Leica, Germany) was used as a light filter for the microscope light. The high-energy UV light was irradiated from a Leica EL6000 external light source (Leica, Germany) connected to the microscope.

**Characterization**

Scanning electron microscopy (SEM) images and energy-dispersive X-ray (EDX) line-scanning analysis were obtained using a Hitachi S-4800 field-emission SEM (Japan). EDX mapping was obtained using a JEOL JEM-2100F field-emission Transmission electron microscopy (TEM) (Japan). The X-ray diffraction (XRD) pattern of the samples was recorded on a Bruker D8 Advance X-ray diffractometer (Germany). The zeta potential of MPs was obtained by NanoBrook 90 Plus Zeta (US). *C*_CO2_ in various water media was calculated from the measured pH value.

**Governing equations for numerical simulations**

When ZnO reacts with the H^+^ dissociated from H_2_CO_3_ in the medium, Zn^2+^ cations and HCO_3_^-^ anions are released from the exposed ZnO surface of the ZnO/SiO_2_ micromotor (MM) due to the corrosion of ZnO and the buffering effect of H_2_CO_3_. The distribution of ionic species *i* (Zn^2+^, H^+^ or HCO_3_^-^) originates from its flux (*J_i_*) from the exposed ZnO surface of the ZnO/SiO_2_ MM, and is further affected by the diffusion, convection and migration of ions (Equation 1). In our model, this was solved with the conservation equation (Equation 2) at a steady state.

$\boldsymbol{J}_{i}=\boldsymbol{u}c_{i}-D_{i}\boldsymbol{\nabla}c_{i}-\frac{z_{i}FD_{i}c_{i}\boldsymbol{\nabla}\varphi}{\mathrm{RT}}$ (1)

$\boldsymbol{\nabla\cdot}\boldsymbol{J}_{i}=\boldsymbol{0}=\boldsymbol{u\cdot}\boldsymbol{\nabla}c_{i}-D_{i}\boldsymbol{\nabla}^{2}c_{i}-\frac{z_{i}FD_{i}\boldsymbol{\nabla}\boldsymbol{\cdot}\left( c_{i}\boldsymbol{\nabla}\varphi\right)}{\mathrm{RT}}$ (2)

Where $\boldsymbol{u}$ is the fluid velocity, $F$ is the Faraday constant, $\varphi$ is the electrostatic potential, R is the gas constant, T is the absolute temperature, and $c_{i}$, $D_{i}$, $z_{i}$ are the concentration, diffusion coefficient, and charge of species *i*, respectively.

The electric potential ($\varphi$) in Equation 1 around the ZnO/SiO_2_ MM is calculated using the Poisson equation,

$\mathbf{-}\varepsilon_{0}\varepsilon_{r}\boldsymbol{\nabla}^{2}\varphi=\rho_{e}=F\left( z_{+}c_{+}+z_{-}c_{-} \right)$ (3)

where $\rho_{e}$ is the volumetric charge density, $z_{+}$ and $z_{-}$ are the charges of the cations and the anions, $c_{+}$ and $c_{-}$ are the concentrations of the cations and the anions, $\varepsilon_{0}$ is the permittivity of the vacuum, and $\varepsilon_{r}$ is the relative permittivity of the fluid media, respectively.

The inertial effect is neglected in the present study because of a very small Reynolds number. Thus, the flow field is governed by the Stokes equations,

$-\nabla p+\mu\nabla^{2}\boldsymbol{u}=0$ (4)

and the continuity equation for the incompressible fluid,

$\nabla\boldsymbol{\cdot}\boldsymbol{u}\mathbf{=}0$ (5)

In these equations, ***u*** is the fluid velocity vector, and *p* is the pressure. The initial values of the flow velocity and the pressure are all zero. The electroosmotic flow boundary condition on the particle surface is as following,

$\boldsymbol{u}= \frac{\varepsilon_{0}\varepsilon_{r}\zeta_{p}}{\mu}\left( \mathbf{I}-\mathbf{nn} \right)\cdot\nabla\varphi$ (6)

In this equation, $\zeta_{p}$ is the zeta potential of the ZnO/SiO_2_ MM. The quantity $\left( \mathbf{I}-\mathbf{nn} \right)$ defines the electric field tangential to the charged surface, with **I** denoting the second-order unit tensor. Through the numerical simulations, the concentrations of Zn^2+^, H^+^ and HCO_3_^-^, local diffusio-electric field *E* and flow field induced by surface electroosmotic slips around the ZnO/SiO_2_ MM were obtained.

To calculate the self-electroosmotic torque *M*_p_, the forces of the flow acting on the surface of the MM (*F_x_* and *F_y_*) were at first decomposed into a force tangential to the motor surface (*F_t_*) and that along the radius direction (*F_r_*),

$F_{t}\text{=}\frac{\text{y}}{\text{r}}\text{F}_{\text{x}}+\frac{\text{x}}{\text{r}}\text{F}_{\text{y}}$ (7)

$F_{r}\text{=}\frac{\text{y}}{\text{r}}\text{F}_{\text{y}}+\frac{\text{x}}{\text{r}}\text{F}_{\text{x}}$ (8)

Then, *M*_p_ was calculated as follow,

$M_{p}\text{=}F_{t}r$ (9)

To analyze the diffusion kinetics of atmospheric CO_2_ molecules into water through the interface with the atmosphere, the classical two-film theory was used,[1]

$\boldsymbol{J}_{\mathrm{CO}_{2}}=k(C^{*}-C)$ (10)

where $\boldsymbol{J}_{\mathrm{CO}_{2}}$ is the flux of atmospheric CO_2_ molecules, *k* is the mass transfer coefficient of CO_2_ in water. *C*^*^ is the equilibrium concentration of CO_2_ at the interface, and *C* is the bulk concentration of CO_2_ in water.

**SUPPLEMENTARY FIGURES**


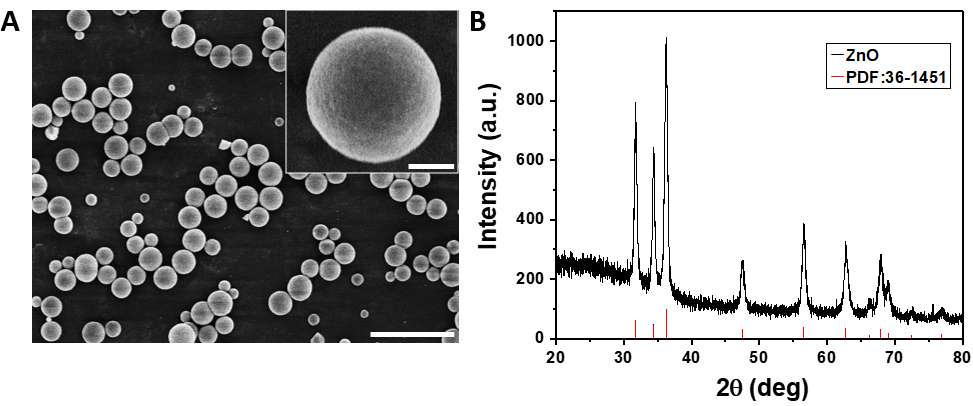


**Supplementary Figure 1.** (A) SEM images and (B) XRD pattern of the ZnO microspheres. Scale bar, 10 μm. Scale bar in the inset of A, 1 μm.


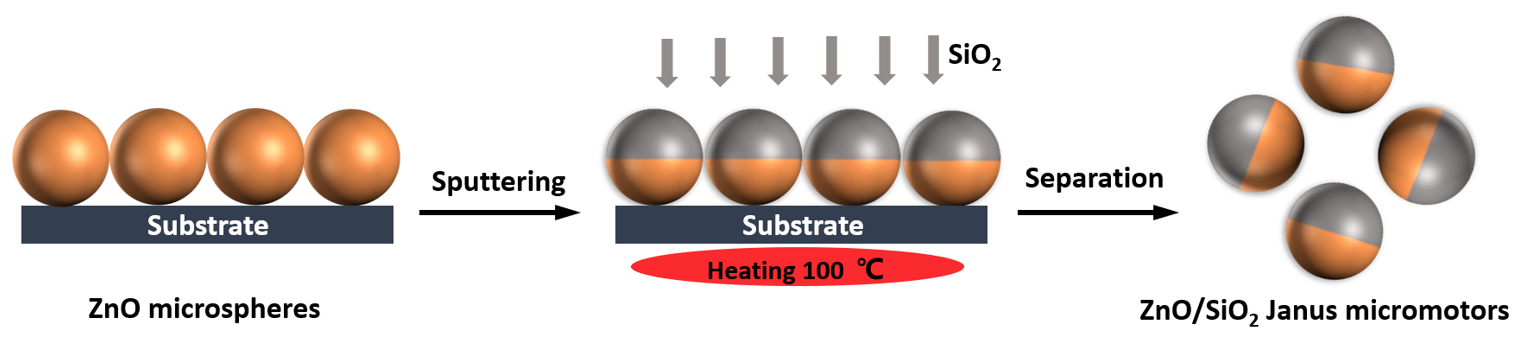


**Supplementary Figure 2.** Schematic illustration of the preparation of the ZnO/SiO_2_ MMs.


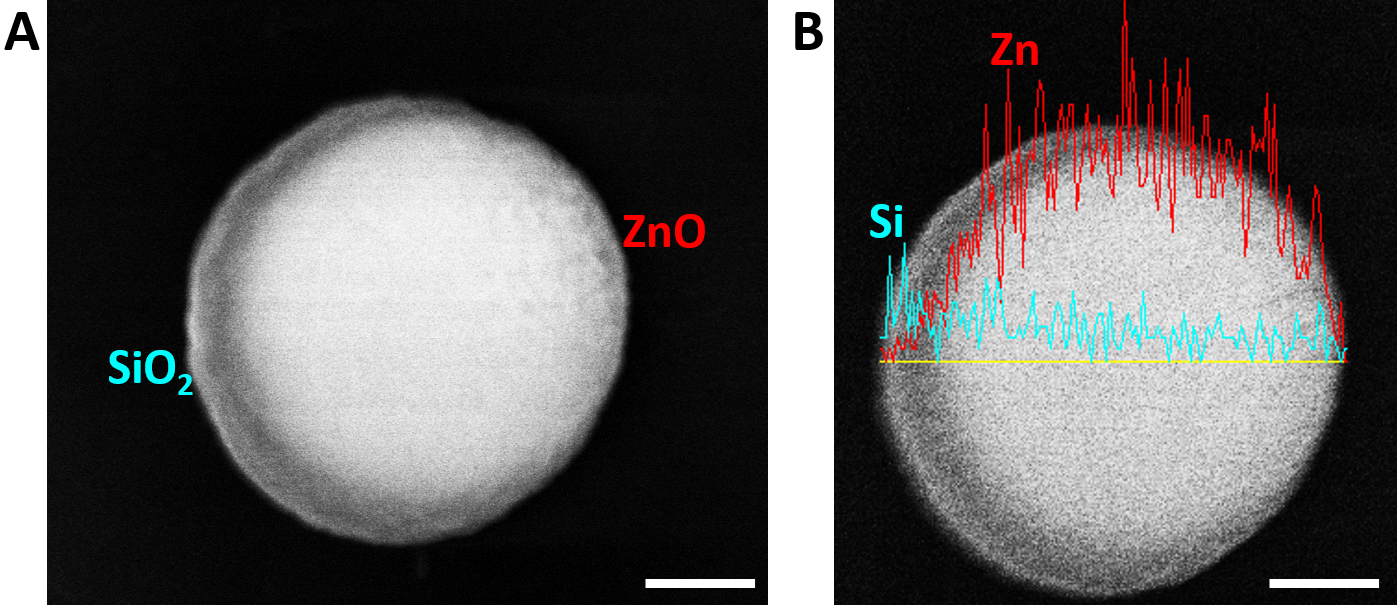


**Supplementary Figure 3.** (A) The high-contrast SEM image and (B) the line-scanning EDX analysis of Si (cyan curve) and Zn (red curve) in a ZnO/SiO_2_ MM. Scale bars, 500 nm.


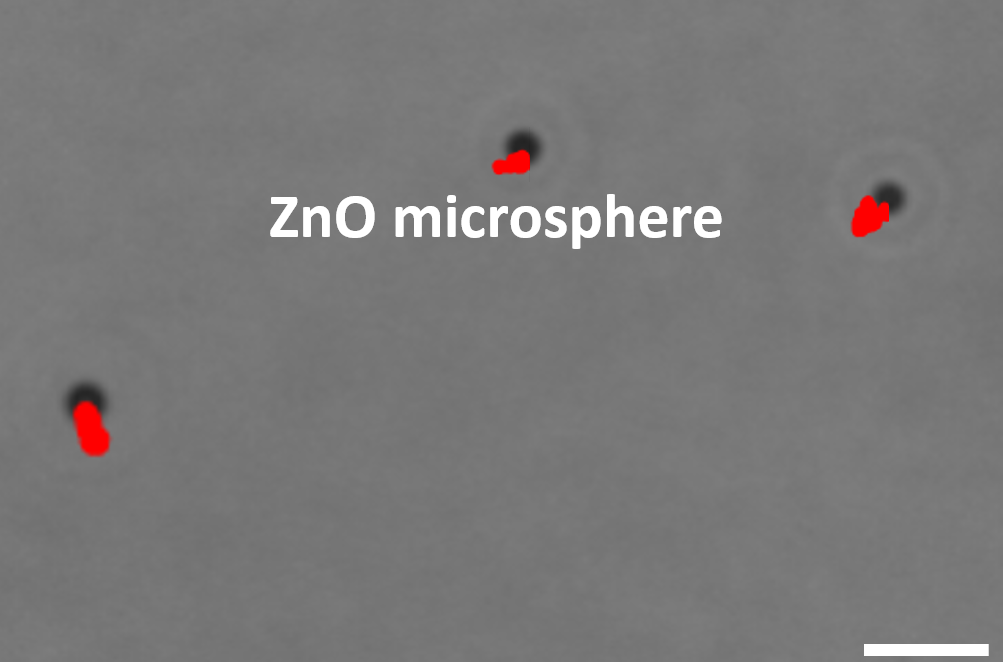


**Supplementary Figure 4.** Trajectories of the ZnO microspheres in the air-exposed water in 5 s. Scale bar, 5 μm.


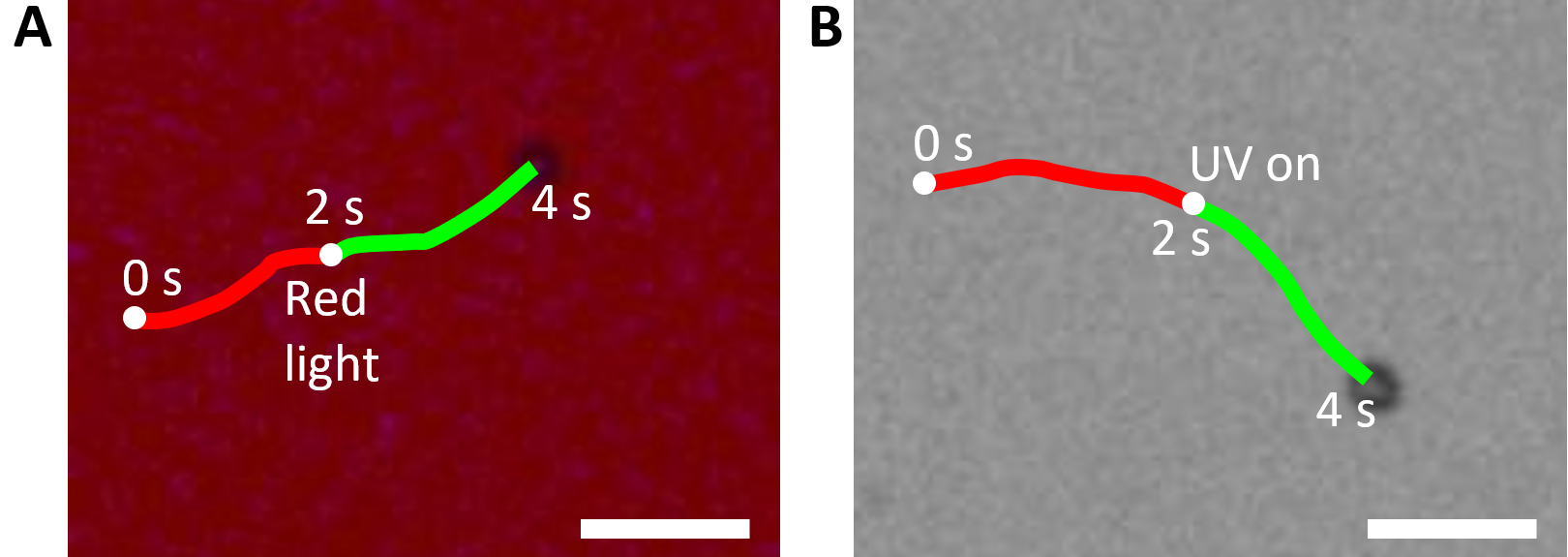


**Supplementary Figure 5.** Trajectories of the ZnO/SiO_2_ MMs under the illumination of (A) the low-energy red light (20.2 mW/cm^2^, green curve) and (B) the high-energy UV light (300 mW/cm^2^, green curve). Red curves are the trajectories of the MMs under the illumination of the microscope. Scale bars, 5μm.


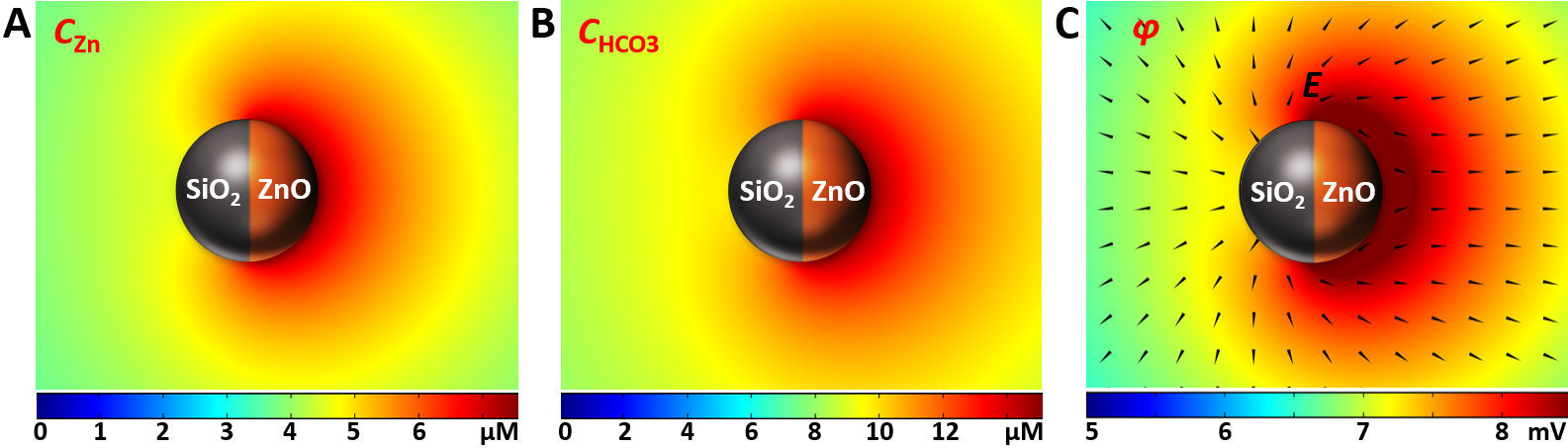


**Supplementary Figure 6.** (A, B) Numerical simulations of the concentration of (A) Zn^2+^ (*C*_Zn_) and (B) HCO_3_^-^ (*C*_HCO3_) around a ZnO/SiO_2_ MM. (C) Numerical simulation of the electric potential (*φ*, the color background) and local diffusion-electric field (*E*, black triangles) around a ZnO/SiO_2_ MM.


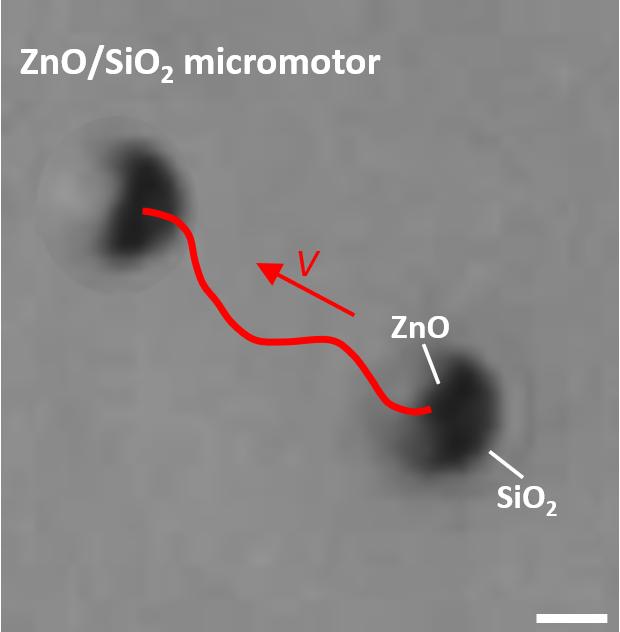


**Supplementary Figure 7.** The highly-magnified microscopic image of an etched ZnO/SiO_2_ MM, suggesting that it moves with the ZnO end forward. The red curve is the trajectory of the MM in 7 s. Scale bar, 1 μm.


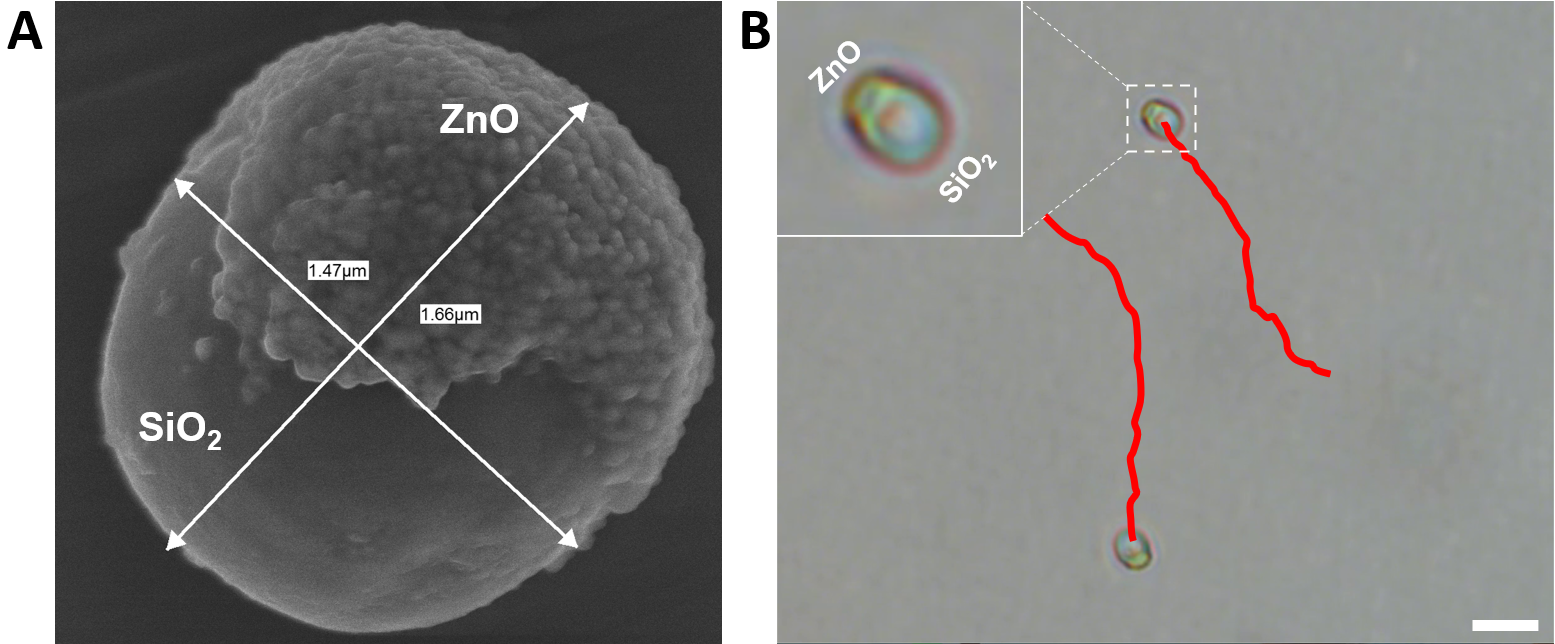


**Supplementary Figure 8.** (A) The SEM image of the SiO_2_/ZnO MM. (B) Trajectories of the SiO_2_/ZnO MMs in the air-exposed water, further suggesting that the ZnO-based MMs move with the ZnO end forward. The red curve is the trajectory of the MM in 6 s. Scale bar, 5 μm.


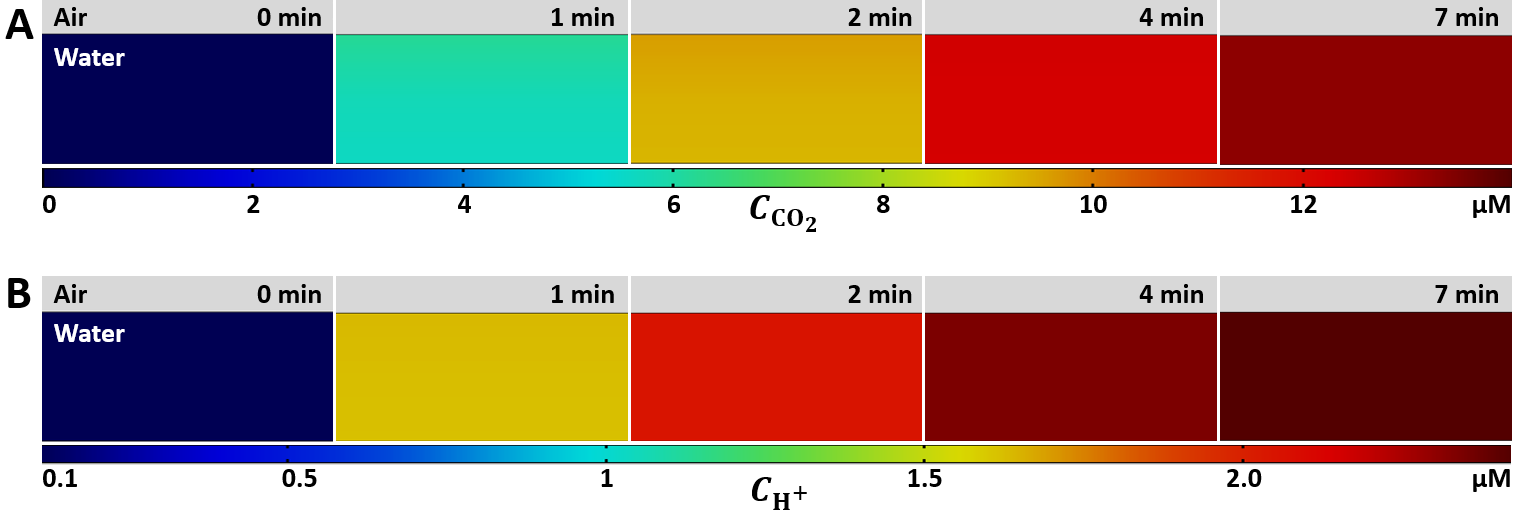


**Supplementary Figure 9.** Visualized color images of the numerical simulations of (A) *C*_CO2_ and (B) $C_{H^{+}}$ in a CO_2_-eliminated water film (160 μm in thickness) as a function of time when exposed to air.


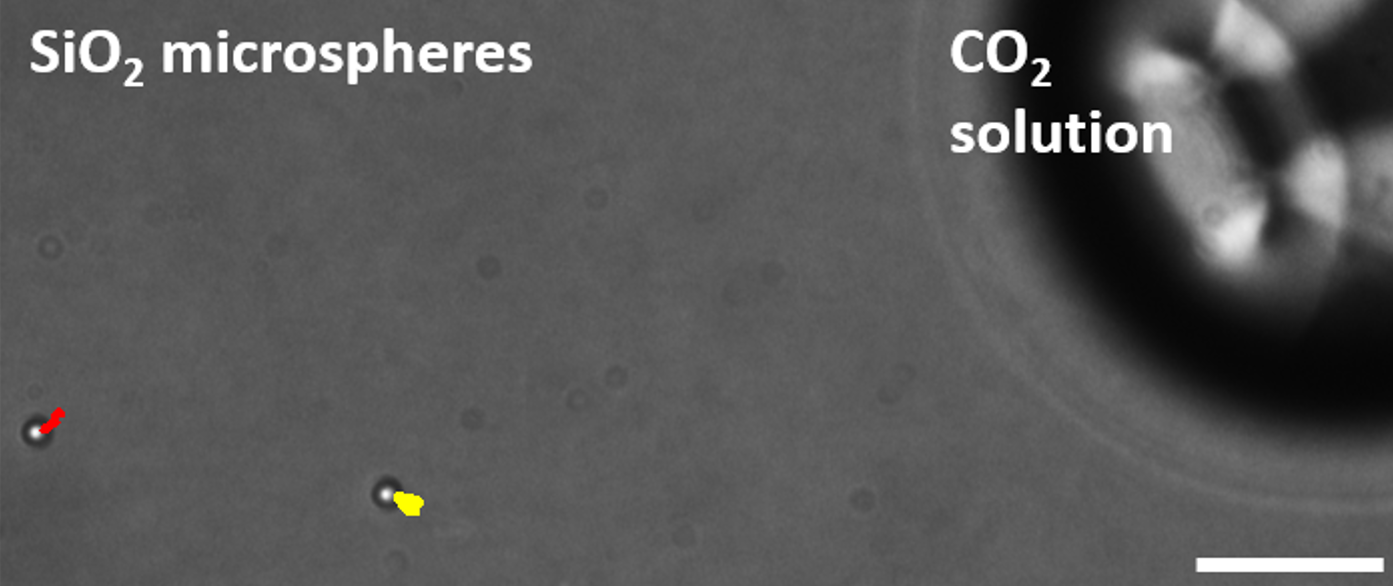


**Supplementary Figure 10.** Trajectories of two passive SiO_2_ microspheres in 40 s around a micropipette filled with the CO_2_ solution with a *C*_CO2_ of 93.7 μM. Scale bar, 20 μm.


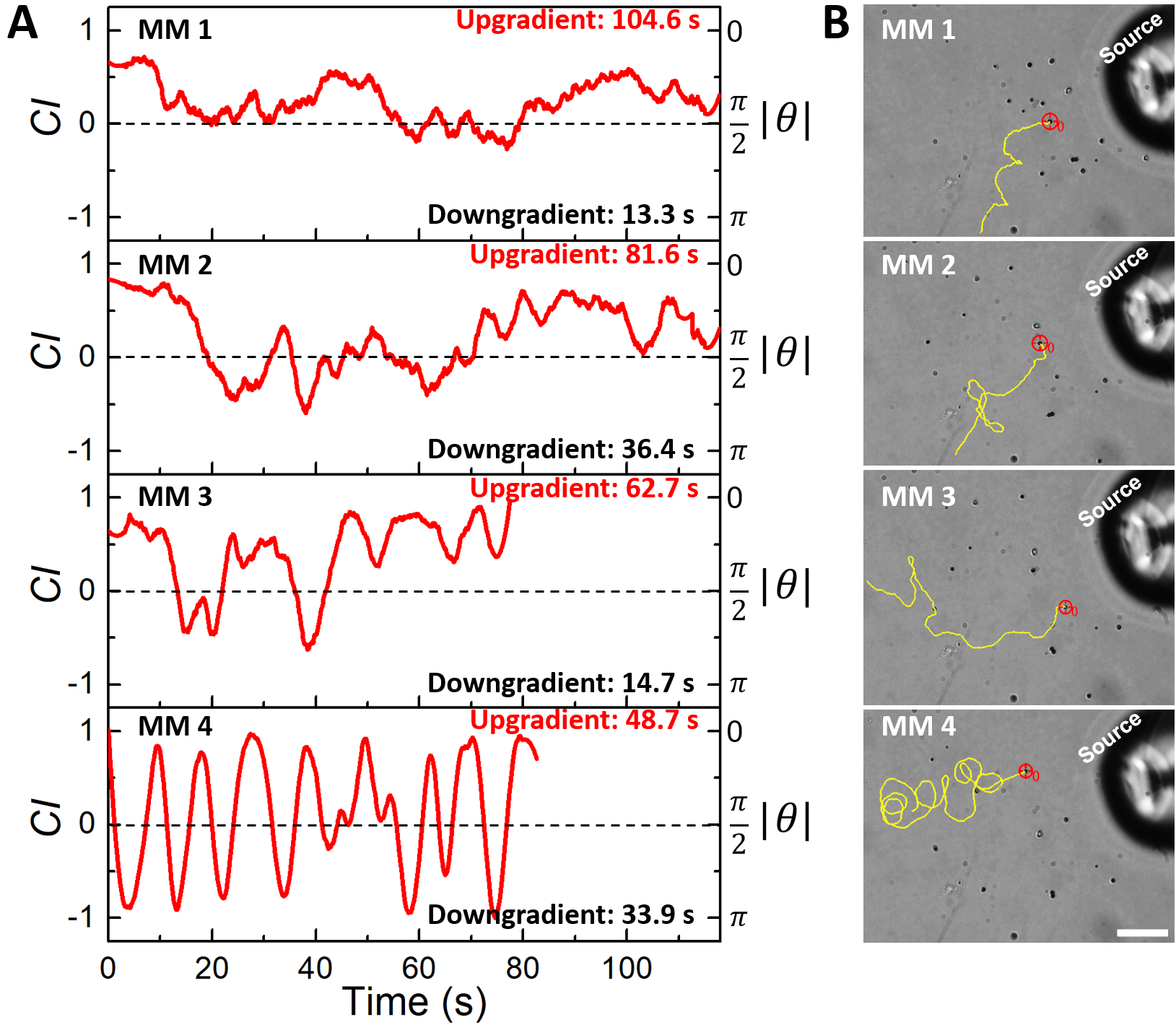


**Supplementary Figure 11** (A) Chemotaxis index (*CI*) and the absolute value of the angle (|*θ*|) between the MM's motion direction and the chemical gradient as a function of time of four typical ZnO/SiO_2_ MMs when they move toward the CO_2_ source. (B) The corresponding trajectories of these four ZnO/SiO_2_ MMs toward the CO_2_ source. Scale bar, 20 μm.


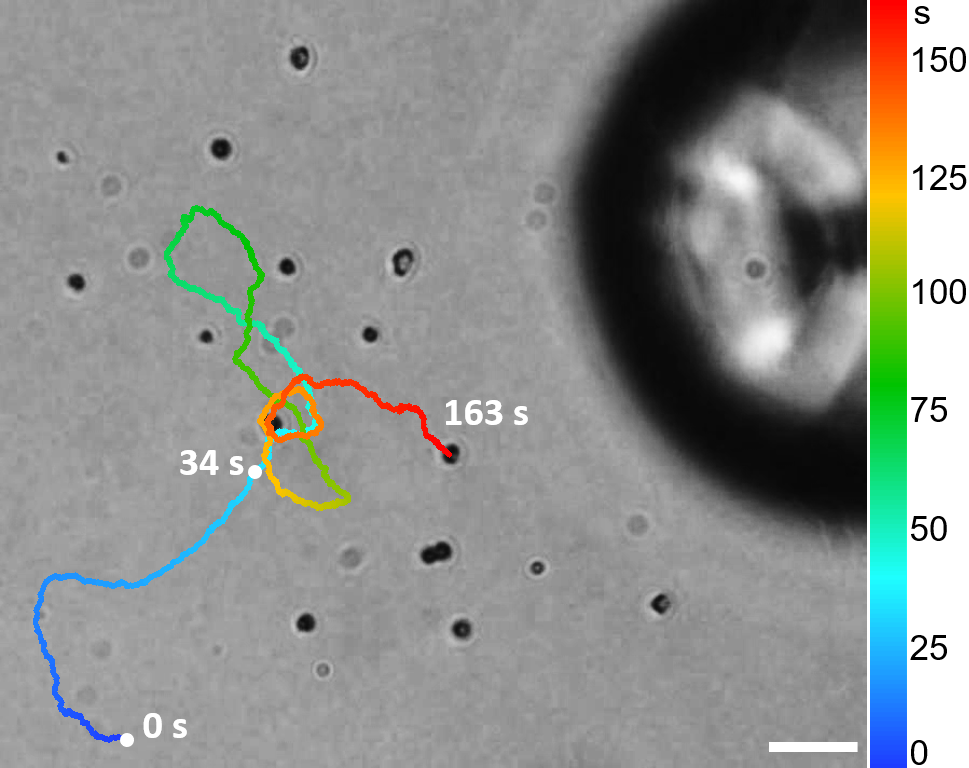


**Supplementary Figure 12.** The trajectory of a slightly etched chemotactic ZnO/SiO_2_ MM moving towards the CO_2_ source in 163 s. Scale bar, 20 μm.

**SUPPLEMENTARY VIDEOS**

**Supplementary Video 1.** Self-propulsions of the ZnO/SiO_2_ MMs in the air-exposed water and the CO_2_-eliminated water.

**Supplementary Video 2.** Self-propulsions of the ZnO-based MMs under light illuminations20.2 mW/cm^2^.

**Supplementary Video 3.** Self-propulsions of a partially etched ZnO/SiO_2_ MM and barely etched SiO_2_/ZnO MMs in the air-exposed water.

**Supplementary Video 4.** Self-propulsions of the ZnO/SiO_2_ MMs in the freshly boiled water over time when exposed to air.

**Supplementary Video 5.** Self-propulsions of the ZnO/SiO_2_ MMs in water with different CO_2_ concentrations.

**Supplementary Video 6.** The positive chemotaxis of the ZnO/SiO_2_ MMs toward the CO_2_ source.

**Supplementary Video 7.** Random Brownian motions of passive SiO_2_ microspheres near the CO_2_ source.

**REFERENCES**

1. Whitman WG. The two film theory of gas absorption. *Int J Heat Mass Tran* 1962; **5**: 429-33.
